# Emerging burdens of adolescent psychosocial health problems: a population-based study of 202 040 adolescents from 68 countries

**DOI:** 10.1192/bjo.2023.583

**Published:** 2023-10-16

**Authors:** Md Ashfikur Rahman, Satyajit Kundu, Enryka Christopher, Bright Opoku Ahinkorah, Joshua Okyere, Riaz Uddin, Rashidul Alam Mahumud

**Affiliations:** Development Studies Discipline, Khulna University, Bangladesh; Global Health Institute, North South University, Bangladesh; and Faculty of Nutrition and Food Science, Patuakhali Science and Technology University, Bangladesh; Trauma and Community Resilience Center, Department of Psychiatry, Boston Children's Hospital, Boston, MA, USA; and Department of Psychiatry, Harvard Medical School, Boston, MA, USA; School of Clinical Medicine, University of New South Wales, Sydney, NSW, Australia; Department of Population and Health, University of Cape Coast, Ghana; School of Health and Rehabilitation Sciences, The University of Queensland, Australia; Health Economics and Health Technology Unit, NHMRC Clinical Trials Centre, Faculty of Medicine and Health, The University of Sydney, Australia

**Keywords:** Epidemiology, low- and middle-income countries, risk assessment, depressive disorders, anxiety or fear-related disorders

## Abstract

**Background:**

Psychosocial health problems are major public health burdens for adolescents. Identifying risk factors is essential to containing negative health implications.

**Aims:**

This study aimed to estimate the burden of psychosocial health problems among adolescents, and identify potential risk and protective factors.

**Method:**

We used the Global School-based Student Health Survey data from 68 countries across six World Health Organization regions. We computed the overall, national- and regional-level weighted estimates of the mean number of psychosocial health problems. Adjusted Poisson regression models examined the factors associated with psychosocial health problems.

**Results:**

Our sample totalled 202 040 adolescents aged 11–17 years (mean age 14.6 [s.d. 1.18] years), composed of 95 589 (47.31%) boys and 104 191 (51.57%) girls (2260 (1.12%) missing answers). Samoa had the highest mean number of psychosocial health problems (mean 2.64 [s.d. 1.41]), and Niue had the lowest (mean 0.33 [s.d. 0.72]). In the pooled analysis, the following factors were associated with higher rates of psychosocial health problems in adolescents globally: ≥13 years of age, being female, experiencing food insecurity, experiencing physical violence, having been bullied, engagement in physical altercation, experiencing serious injury, missing school without parental permission, lack of parental support or monitoring, parents who were not understanding of their child's problems and high sedentary behaviour. Being female, food insecurity, bullying, physical attacks or serious injury were all significantly associated with higher rates of psychosocial health problems in each of the six regions separately.

**Conclusions:**

The prevalence of psychosocial health problems is high among adolescents, although there are country-level variations. Health promotion strategies should consider the identified factors to promote adolescents’ health and well-being.

Globally, in 2017, mental health disorders or psychosocial health problems were the second leading cause of disease burden in terms of years lived with disability, and the sixth leading cause of disability-adjusted life-years.^1^ Poor mental health presents a significant challenge to healthcare systems, especially in low- and middle-income countries (LMICs).^1^ Mental health has been included in the United Nations Sustainable Development Goals target 3.4, ‘Noncommunicable diseases and mental health’.^2^ Similarly, the launch of the World Health Organization's (WHO) Mental Health Action Plan 2013–2020^3^ also played a significant role in tracking progress with respect to remediating psychosocial health problems and other mental health disorders.^4^ Nevertheless, psychosocial health problems continue to affect the majority of the world's population, including adolescents.

As adolescence is a time of substantial change and transition, it is associated with a high prevalence of psychosocial health disorders.^5^ Evidence suggests that 10–20% of all adolescents experience at least one form of psychosocial health problem. However, most cases of psychosocial health problems in adolescents go undetected and untreated.^6,7^ Notably, depression and generalised anxiety disorders are the most common psychosocial health problems among adolescents.^8^ Between 1992 and 2017, there has been a 70% increase in global levels of anxiety and depression among adolescents.^9^ Apart from depression and anxiety, loneliness is also a recurring psychosocial health problem among adolescents. A study in Denmark reported that the prevalence of loneliness in adolescents increased from 4.4% in 1991 to 7.2% in 2014. Psychosocial health problems have several concomitant effects on the health outcomes of adolescents.^10^ Related studies have found that psychosocial health problems among adolescents heighten the risk of impaired social relations, substance misuse, school drop-out and suicide.^11,12^ Some studies have found factors such as being female, being bullied, age and lack of social support are associated with adolescent psychosocial health problems.^13,14^ Yet, variations at global and regional levels remain unclear.

Although psychosocial health problems often co-occur, few studies have investigated the occurrence of multiple psychosocial health problems among adolescents on a global scale. In addition, little is known about the potential risk and protective factors of multiple psychosocial health problems in adolescents. A large-scale, comprehensive population-based study of adolescent psychosocial health and associated risk and protective factors at a global, regional and national level is warranted. Estimating the burden of psychosocial health problems among adolescents is necessary to inform relevant programmes, policies and strategies to remediate them. Extant studies^13,15,16^ have mainly investigated the burden of psychosocial health problems in adolescents at the country level, with global and regional dynamics receiving little to no attention. This limitation in existing literature impedes development of regional and global interventions to reduce the prevalence of psychosocial health problems. Findings from this study can help to inform such preventive programmes and project future trends.

## Aims

The main aims of this study were to measure the burden of psychosocial health problems among adolescents and identify associated factors.

## Method

### Study design and data source

This study used data from the Global School-based Student Health Survey (GSHS), collected between 2003 and 2015. All publicly available, nationally representative data-sets that included variables pertaining to the analysis were selected for this study. For countries with more than one GSHS data-set, the most recent data-set available was used. Country-level response rates were determined based on school and student response rates.^17^ Participants included school-going children aged 11–17 years representing both LMICs and high-income countries (HICs). In all participating countries, the GSHS collected data with the same measures, some of which were adapted to match country-specific sociocultural contexts.^17^ Countries adapted questionnaires by combining standardised core questions with country-specific examples, options or phrases. The surveys were translated into the students’ native language and pilot tested to ensure understandability. A two-stage cluster sampling technique was employed in collecting data for the GSHS to obtain a representative sample of students across participating countries. Schools from a wide range of geographic regions in each country were selected first. A sample of these schools was chosen based on probability proportional to enrolment size, after which classes within chosen schools were randomly selected such that all students had an equal probability of participation. All students within selected classes were eligible. The survey was administered during class time.^17^ Detailed information regarding survey sampling, quality control, management and survey instruments are available from the WHO.^17^ Surveys included questions on demographics, hunger, peer victimisation, loneliness, anxiety, peer conflict or violence, substance use, parental support and behaviours, and lifestyle factors such as sedentary behaviour.

The GSHS received ethics approval from the participating country's ethics governing bodies, such as the Ministry of Education or other appropriate institutional ethics review committees. Written or verbal consent of parents was required to participate. Participation in the survey was anonymous.

### Measures

#### Outcome variable: psychosocial health problems

The simplest approach to investigate the magnitude of a country's mental health burden is to measure the number of psychosocial health problems reported.^14,18,19^ Thus, this study used the reported number of psychosocial health problems as an outcome variable. A simple count of each condition after dichotomisation was used to measure psychosocial health problems; for example, loneliness was assessed with ‘How often have you felt lonely during the past 12 months?’, and anxiety was assessed with ‘During the past 12 months, how often have you been so worried about something that you could not sleep at night?’. Response options were ‘never’, ‘rarely’, ‘sometimes’, ‘most of the time’ and ‘always’. The answers ‘most of the time’ and ‘always’ were recoded as ‘yes’, whereas ‘never’, ‘rarely’ and ‘sometimes’ were characterised as ‘no’, to form binary variables. Suicidal ideation, planning and attempt were already binary variables (e.g. ‘Did you ever seriously consider attempting suicide during the past 12 months?’ with answers of ‘yes’ or ‘no’). If any adolescent responds affirmatively to any of the aforementioned questions on psychological health problems, we included them in our final analytical exploration.

### Independent variables

#### Sociodemographic characteristics

The gender of participants was coded as male or female. Age was a continuous variable. Food insecurity was measured with ‘During the past 30 days, how often did you go hungry because there was not enough food in your home?’. Responses of ‘most of the time’ or ‘always’ were recoded as having severe food insecurity, ‘rarely’ or ‘sometimes’ as moderate food insecurity and ‘never’ as no food insecurity.

#### Peer victimisation and conflict

Violence and other peer conflict were assessed by asking about frequencies and intensity of physical injuries, participation in physical altercations and bullying. Students were asked to report the number of times they were physically attacked and/or in physical altercations during the past 12 months. All responses were dichotomised, with those who reported one or more instances of any event recoded as ‘yes’.

#### Peer and parental support

Peer support at school, parental understanding, parental monitoring and parental control as perceived by the adolescent were measures of social support. Peer support was assessed by asking ‘During the past 30 days, how often were most of the students in your school kind and helpful?’. Three questions were used to assess parent–adolescent relationships: ‘During the past 30 days, how often did your parents or guardians understand your problems and worries?’; ‘During the past 30 days, how often did your parents or guardians really know what you were doing with your free time?’ and ‘How often did your parents or guardians go through your things without your approval during the past 30 days?’. Responses for each item were ‘never’, ‘rarely’, ‘sometimes’, ‘most of the time’ and ‘always’. These were recoded to trichotomous variables of ‘never/rarely’, ‘sometimes’ and ‘most of the time/ always’.

#### Health and lifestyle factors

Health and lifestyle factors included questions on food insecurity, sedentary activities and obesity. Participants were asked about time spent engaged in stationary activities, along with their weight and height. Students were asked ‘How much time do you spend during a typical or usual day sitting and watching television, playing computer games, talking with friends, or doing other sedentary activities?’. Answer choices were none, <1 h, 1–2 h, 3–4 h and ≥5 h. Height and weight data were self-reported rather than measured. Body mass index (BMI) was calculated for each participant by dividing weight by height squared (kg/m^2^). Individual BMIs were then compared with age- and gender-specific *z-*scores, using the 2007 WHO standards.^20^ Study participants were classified as having normal BMI if their calculated score fell within 2 s.d. below or 1 s.d. above the WHO mean *z*-score, overweight was defined as falling above 1 s.d. and obese as above 2 s.d.

### Statistical analyses

Weighted estimates of the mean number of psychosocial health problems were calculated with corresponding 95% confidence intervals for the national and regional data. Sampling weights were used to make the estimate nationally representative. Because of the complex nature of the data, the option of a composite sample was applied in the analytical exploration, accounting for the country-specific primary sampling unit (PSU), stratum, and sample weight. All analyses were weighted using a PSU derived from the probability of a school being selected, a classroom being selected, school- and student-level non-response, grade and gender. This included using strata and PSU with country-specific data. The dependent variable (i.e. reported number of psychosocial health problems) was characterised as under-dispersed property of a count measure (variance < mean). In the analytical exploration, adjusted generalised Poisson regression models were employed under the satisfaction of an under-dispersion assumption, to examine the association between the magnitude of psychosocial health problems and other variables. All of the analyses performed in this study were conducted with statistical software Stata version 16.0 for Windows (StataCorp, College Station, Texas, USA).

## Results

### Sample characteristics

A total of 202 040 adolescents aged 11–17 years from 68 LMICs and HICs were included in this study; 95 589 (47.31%) were boys and 104 191 (51.57%) were girls, with 2260 (1.1%) missing responses. More than a fourth of the adolescents had experienced bullying (28.12%) and physical attacks (27.39%). Almost a third of respondents were involved in physical altercations (34.60%) and suffered serious injury (32.48%). Almost 40% of adolescents reported never having peer support, and 38.27% reported that their parents never understood their problems. We also found that 43.93% of adolescents were not monitored by guardians, and about 11.27% missed school without permission. In this study, 15.66% of participants were either overweight (10.97%) or obese (4.69%) (Table 1).

**Table 1:** Mean number of psychosocial health problems by participant characteristics

Variable	*n* (%)	Mean (s.d.)	95% CI
Age in years			
11–12 years	16 449 (8.14)	1.50 (1.15)	1.48–1.51
13 years	37 650 (18.63)	1.56 (1.19)	1.55–1.57
14 years	50 295 (24.89)	1.67 (1.21)	1.66–1.68
15 years	47 375 (23.45)	1.77 (1.23)	1.76–1.78
16 years	35 147 (17.40)	1.91 (1.26)	1.89–1.92
17 years	13 279 (6.57)	1.93 (1.25)	1.90–1.95
Missing response	1845 (0.91)		
Gender of adolescent			
Boy	95 589 (47.31)	1.57 (1.17)	1.56–1.57
Girl	104 191 (51.57)	1.85 (1.26)	1.85–1.86
Missing response	2260 (1.12)		
Food insecurity			
None	102 605 (50.78)	1.52 (1.19)	1.51–1.52
Sometimes or rarely	73 878 (36.57)	1.95 (1.22)	1.94–1.96
Most of the time or always	13 460 (6.66)	2.15 (1.34)	2.12–2.17
Missing response	12 097 (5.99)		
Bullied			
No	120 098 (59.44)	1.51 (1.16)	1.51–1.52
Yes	56 816 (28.12)	2.16 (1.30)	2.215–2.17
Missing response	25 126 (12.44)		
Physically attacked			
No	108 578 (53.74)	1.60 (1.21)	1.59–1.61
Yes	55 341 (27.39)	2.00 (1.34)	1.99–2.02
Missing response	38 121 (18.87)		
Physical altercation			
No	127 523 (63.12)	1.62 (1.18)	1.62–1.3
Yes	69 912 (34.60)	1.91 (1.29)	1.90–1.92
Missing response	4605 (2.28)		
Seriously injured			
No	95 181 (47.11)	1.52 (1.16)	1.51–1.52
Yes	65 624 (32.48)	2.02 (1.30)	2.01–2.03
Missing response	41 235 (20.41)		
Peer supports			
Never	79 338 (39.27)	1.59 (1.19)	1.58–1.60
Sometimes or rarely	28 569 (14.14)	1.75 (1.32)	1.73–1.76
Most of the time or always	91 113 (45.1)	1.82 (1.22)	1.82–1.83
Missing response	3020 (1.49)		
Parents check homework			
Never	78 641 (38.92)	1.55 (1.19)	1.54–1.56
Sometimes or rarely	48 299 (23.91)	1.87 (1.30)	1.86–1.88
Most of the time or always	72 318 (35.79)	1.80 (1.19)	1.79–1.81
Missing response	2782 (1.38)		
Parents understand their child's problems			
Never	77 327 (38.27)	1.51 (1.15)	1.50–1.52
Sometimes or rarely	46 745 (23.14)	1.85 (1.35)	1.84–1.86
Most of the time or always	74 666 (36.96)	1.85 (1.19)	1.84–1.86
Missing response	3302 (1.63)		
Parent monitoring			
Never	88 766 (43.93)	1.55 (1.16)	1.54–1.55
Sometimes or rarely	38 423 (19.02)	1.85 (1.35)	1.84–1.86
Most of the time or always	70 597 (34.94)	1.87 (1.21)	1.86–1.87
Missing response	4254 (2.11)		
Miss school without permission			
No	179 270 (88.73)	1.68 (1.20)	1.67–1.68
Yes	22 770 (11.27)	2.04 (1.36)	2.03–2.06
Missing response	0 (0.00)		
Sedentary activities per day			
<1 h	62 855 (31.11)	1.62 (1.20)	1.61–1.62
1–2 h	60 496 (29.94)	1.65 (1.18)	1.64–1.66
3–4 h	36 558 (18.09)	1.77 (1.22)	1.76–1.79
>4 h	32 272 (15.97)	1.95 (1.31)	1.94–1.96
Missing response	9859 (4.88)		
Adolescent BMI			
Normal	170 407 (84.34)	1.72 (1.22)	1.72–1.73
Overweight	22 159 (10.97)	1.67 (1.26)	1.65–1.69
Obese	9472 (4.69)	1.77 (1.26)	1.74–1.80
Missing response	2 (0.00)		

BMI, body mass index.

Among 68 countries, the highest mean [s.d.] value of psychosocial health problems was found in Samoa (2.64 [1.41]), followed by the Solomon Islands (2.55 [1.36]) and Benin (2.29 [1.45]). In contrast, Niue (0.33 [0.72]), Egypt (0.62 [0.57]) and Vietnam (0.96 [0.67]) all had lower mean values of psychosocial health problems than other countries (Table 2).

**Table 2:** Distribution of psychosocial health problems among school-going adolescents, by country

Country	Number of participants	Number of psychosocial health problems			
		Mean (s.d.)	95% CI	Median	IQR
Afghanistan	2465	1.88 (1.20)	1.83–1.93	2	1
Antigua and Barbuda	1188	1.79 (1.39)	1.71–1.87	2	1
Argentina	27 205	1.78 (1.38)	1.76–1.80	2	1
Bahamas	1294	1.66 (1.36)	1.59–1.73	1	1
Bangladesh	2882	1.43 (1.01)	1.39–1.47	2	1
Barbados	1602	1.15 (0.79)	1.11–1.19	1	1
Belize	2081	1.74 (1.35)	1.68–1.80	2	1
Benin	2684	2.29 (1.45)	2.23–2.34	2	2
Bolivia	3666	2.06 (1.36)	2.02–2.11	2	2
Botswana	2149	2.02 (1.11)	1.97–2.06	2	2
British Virgin Islands	1631	1.66 (1.30)	1.59–1.72	1	1
Brunei Darussalam	2558	1.73 (1.01)	1.69–1.77	2	1
Cayman Islands	1191	1.80 (0.86)	1.75–1.85	2	1
China	8902	1.75 (0.85)	1.73–1.77	2	1
Cook Islands	695	1.73 (1.36)	1.63–1.83	2	1
Costa Rica	2647	1.47 (1.19)	1.42–1.51	1	1
Curacao	2628	1.60 (1.25)	1.56–1.65	2	1
Djibouti	1737	1.49 (0.99)	1.44–1.53	2	1
Ecuador	5384	1.67 (0.91)	1.64–1.69	2	1
Egypt	2529	0.62 (0.57)	0.60–0.64	1	1
El Salvador	1873	1.60 (1.36)	1.54–1.66	1	1
Eswatini	3589	1.86 (1.35)	1.82–1.91	2	2
Ghana	3577	2.16 (1.36)	2.12–2.21	2	2
Grenada	1426	1.85 (0.95)	1.80–1.90	2	1
Guyana	2313	1.92 (1.19)	1.87–1.97	2	2
Honduras	1740	1.81 (1.42)	1.74–1.87	2	1
India	7818	1.40 (0.81)	1.39–1.42	2	1
Iraq	1998	1.80 (1.35)	1.74–1.86	2	1
Jamaica	1584	1.99 (1.40)	1.92–2.06	2	2
Jordan	2128	1.82 (1.21)	1.77–1.87	2	2
Kenya	3384	1.83 (0.97)	1.80–1.86	2	1
Kiribati	1567	2.09 (1.58)	2.01–2.17	2	2
Kuwait	3381	1.93 (1.26)	1.89–1.97	2	1
Lao People's Democratic Republic	3669	1.44 (0.95)	1.41–1.47	2	1
Lebanon	2191	1.60 (1.30)	1.55–1.65	1	1
Mauritania	1996	1.73 (1.28)	1.67–1.79	2	1
Mongolia	5371	1.82 (1.35)	1.78–1.85	2	1
Montserrat	207	1.79 (0.88)	1.67–1.91	2	1
Morocco	2859	1.66 (1.34)	1.61–1.71	2	1
Mozambique	1875	1.87 (1.24)	1.81–1.93	2	1
Myanmar	2788	1.31 (0.58)	1.29–1.33	1	1
Namibia	4451	2.15 (1.35)	2.11–2.19	2	2
Nauru	530	2.01 (1.11)	1.91–2.10	2	2
Niue	135	0.33 (0.72)	0.20–0.45	0	0
Peru	2859	2.15 (1.29)	2.11–2.20	2	2
The Philippines	5241	2.12 (1.03)	2.09–2.14	2	0
Qatar	1807	1.32 (0.82)	1.28–1.36	1	1
Republic of North Macedonia	1982	1.50 (1.01)	1.45–1.54	2	1
Saint Kitts	1700	1.79 (1.31)	1.73–1.85	2	1
Samoa	2050	2.64 (1.41)	2.58–2.70	3	2
Seychelles	2346	1.94 (1.41)	1.88–1.99	2	2
Solomon Islands	1353	2.55 (1.36)	2.48–2.62	2	2
Suriname	1648	1.65 (1.33)	1.58–1.71	2	1
Syria	3055	1.34 (0.80)	1.31–1.37	1	1
Thailand	5762	1.75 (1.22)	1.72–1.78	2	1
Trinidad and Tobago	2726	1.54 (1.36)	1.49–1.59	1	2
Tunisia	2832	1.02 (0.94)	0.99–1.06	1	1
Tuvalu	904	1.48 (1.09)	1.41–1.55	1	1
Uganda	3155	1.73 (1.13)	1.69–1.77	2	1
United Arab Emirates	2506	1.86 (1.24)	1.81–1.91	2	1
United Republic of Tanzania	3757	1.24 (1.18)	1.20–1.28	1	2
Uruguay	3480	1.39 (1.04)	1.36–1.43	1	1
Vanuatu	1099	1.82 (1.26)	1.75–1.90	2	2
Venezuela	2240	1.35 (1.08)	1.31–1.40	1	1
Vietnam	3323	0.96 (0.67)	0.94–0.98	1	0
Wallis and Futuna	1030	1.89 (1.45)	1.80–1.98	2	2
Zambia	1884	1.95 (0.98)	1.90–1.99	2	2
Zimbabwe	3733	1.95 (1.16)	1.91–1.98	2	2
Total	202 040	1.67 (1.24)	1.67–1.68	2	1

IQR, interquartile range (difference between the 75th and 25th percentiles of the data).

### Global factors associated with psychosocial health problems

The adjusted regression model displays an increase in psychosocial health problems as age increases. Girls had a 30% higher rate of experiencing psychosocial health problems than boys (adjusted incidence rate ratio (aIRR) 1.30, 95% CI 1.29–1.31). Experiencing severe food insecurity was a significant predictor of the rate of psychosocial health problems compared with those with food security (aIRR = 1.24, 95% CI 1.21–1.26). Among factors measuring violence, adolescents who experienced bullying (aIRR = 1.27, 95% CI 1.26–1.29), physical attacks (aIRR = 1.08, 95% CI 1.07–1.09), physical altercations (aIRR = 1.08, 95% CI 1.07–1.09) and serious injuries (aIRR = 1.20, 95% CI 1.19–1.22) were associated with higher rates of psychosocial health problems than those who did not have those experiences. Similarly, adolescents who reported that their parents never understood their problems (aIRR=1.11, 95% CI 1.09–1.12) and never monitored their activities (aIRR = 1.07, 95% CI 1.06–1.09) had higher rates of psychosocial health problems. Adolescents who missed school without permission also had higher rates of psychosocial health problems compared with their counterparts (aIRR = 1.08, 95% CI 1.07–1.10). As the length of daily sedentary activities increased, the rate of psychosocial health problems experienced also increased (>4 h: aIRR = 1.16, 95% CI 1.15–1.18). Curiously, obese participants experienced a slightly lower rate of psychosocial health problems compared with those categorised as normal weight, although this statistic was lower in significance than all other calculations (aIRR = 0.98, 95% CI 0.96–1.00; *P* = 0.048) (Table 3).

**Table 3:** Regression analysis showing different factors associated with psychosocial health problems among study participants globally (*N* = 202 040)

	Unadjusted		Adjusted	
	IRR (95% CI)	*P*-value	aIRR (95% CI)	*P*-value
Age in years				
11– 12	1		1	
13	1.04 (1.03–1.06)	<0.0001	1.05 (1.02–1.07)	<0.0001
14	1.12 (1.10–1.14)	<0.0001	1.11 (1.09–1.13)	<0.0001
15	1.19 (1.17–1.20)	<0.0001	1.18 (1.16–1.20)	<0.0001
16	1.27 (1.26–1.29)	<0.0001	1.26 (1.23–1.28)	<0.0001
17	1.29 (1.27–1.31)	<0.0001	1.26 (1.23–1.29)	<0.0001
Gender of adolescent				
Boy	1		1	
Girl	1.18 (1.18–1.19)	<0.0001	1.30 (1.29–1.31)	<0.0001
Food insecurity				
None	1		1	
Sometimes or rarely	1.29 (1.28–1.30)	<0.0001	1.18 (1.17–1.19)	<0.0001
Most of the time or always	1.42 (1.40–1.43)	<0.0001	1.24 (1.21–1.26)	<0.0001
Bullied				
No	1		1	
Yes	1.43 (1.42–1.44)	<0.0001	1.27 (1.26–1.29)	<0.0001
Physically attacked				
No	1		1	
Yes	1.25 (1.25–1.26)	<0.0001	1.08 (1.07–1.09)	<0.0001
Physical altercation				
No	1		1	
Yes	1.18 (1.17–1.18)	<0.0001	1.08 (1.07–1.09)	<0.0001
Seriously injured				
No	1		1	
Yes	1.34 (1.33–1.35)	<0.0001	1.20 (1.19–1.22)	<0.0001
Peer support				
Sometimes or rarely	1		1	
Never	0.96 (0.95–0.97)	<0.0001	0.96 (0.95–0.98)	<0.0001
Most of the time or always	0.87 (0.87–0.88)	<0.0001	0.94 (0.93–0.95)	<0.0001
Parents check homework				
Never	1.20 (1.19–1.22)	<0.0001	1.07 (1.05–1.08)	<0.0001
Sometimes or rarely	1.16 (1.15–1.17)	<0.0001	1.02 (1.01–1.04)	<0.0001
Most of the time or always	1		1	
Parents understand their child's problems				
Never	1.23 (1.22–1.24)	<0.0001	1.11 (1.09–1.12)	<0.0001
Sometimes or rarely	1.23 (1.22–1.24)	<0.0001	1.10 (1.09–1.12)	<0.0001
Most of the time or always	1		1	
Parent monitoring				
Never	1.20 (1.19–1.21)	<0.0001	1.07 (1.06–1.09)	<0.0001
Sometimes or rarely	1.21 (1.20–1.22)	<0.0001	1.08 (1.07–1.09)	<0.0001
Most of the time or always	1		1	
Miss school without permission				
No	1		1	
Yes	1.22 (1.21–1.23)	<0.0001	1.08 (1.07–1.10)	<0.0001
Sedentary activities (hours per day)				
<1 h	1		1	
1–2 h	1.02 (1.01–1.03)	<0.0001	1.04 (1.03–1.06)	<0.0001
3–4 h	1.10 (1.09–1.11)	<0.0001	1.11 (1.09–1.12)	<0.0001
>4 h	1.21 (1.20–1.22)	<0.0001	1.16 (1.15–1.18)	<0.0001
Adolescent BMI				
Normal	1		1	
Overweight	0.97 (0.96–0.98)	<0.0001	1.00 (0.98–1.01)	0.735
Obese	1.03 (1.01–1.04)	<0.0001	0.98 (0.96–1.00)	0.048

IRR, incidence rate ratio; aIRR, adjusted incidence rate ratio; BMI, body mass index.

### Regional factors associated with psychosocial health problems

Table 4 displays the adjusted IRR of psychosocial health problems for six regions of the world. Our findings demonstrate that factors associated with psychosocial health problems vary significantly between these six regions. Being female, experiencing food insecurity, bullying, physical attacks and serious injury were all significantly associated with higher rates of psychosocial health problems in all six regions. Adolescents involved in physical altercations reported higher rates of psychosocial health problems in all regions except Europe, where the finding was not significant. Results also indicated that participants who had peer support (responses of most of the time or always) reported lower rates of psychosocial health problems in all regions, except the Eastern Mediterranean region. Adolescents without parental monitoring reported higher rates of psychosocial health problems in all regions except for Africa and Europe. Adolescents who missed school without permission were associated with higher rates of psychosocial health problems in all regions except Europe. Europe also did not show a significant association between increased psychosocial health problems and sedentary activities of >4 h (Table 4).

**Table 4:** Regional regression analysis (adjusted) showing different factors associated with psychosocial health problems among study participants (*N* = 202 040)

	Africa		The Americas		South-East Asia		Europe		Eastern Mediterranean		Western Pacific	
Characteristics	aIRR (95% CI)	*P*-value	aIRR (95% CI)	*P*-value	aIRR (95% CI)	*P*-value	aIRR (95% CI)	*P*-value	aIRR (95% CI)	*P*-value	aIRR (95% CI)	*P*-value
Age in years												
11–12 (reference)												
13	1.06 (1.00–1.13)	0.043	1.03 (1.00–1.06)	0.075	1.00 (0.92–1.09)	0.940	1.00 (0.76–1.30)	0.975	1.13 (1.07–1.19)	<0.0001	1.01 (0.96–1.06)	0.793
14	1.11 (1.05–1.17)	<0.0001	1.08 (1.05–1.12)	<0.0001	1.04 (0.96–1.13)	0.335	1.23 (0.95–1.59)	0.123	1.26 (1.20–1.33)	<0.0001	1.02 (0.97–1.07)	0.415
15	1.19 (1.12–1.25)	<0.0001	1.15 (1.11–1.18)	<0.0001	1.10 (1.01–1.19)	0.033	1.26 (0.97–1.63)	0.077	1.39 (1.32–1.47)	<0.0001	1.06 (1.01–1.11)	0.012
16	1.30 (1.24–1.37)	<0.0001	1.21 (1.18–1.25)	<0.0001	1.12 (1.02–1.23)	0.021	1.35 (1.04–1.74)	0.025	1.51 (1.42–1.59)	<0.0001	1.12 (1.07–1.17)	<0.0001
17	1.36 (1.29–1.43)	<0.0001	1.23 (1.15–1.30)	<0.0001	1.17 (1.07–1.29)	0.001	1.47 (1.01–2.15)	0.045	1.61 (1.50–1.72)	<0.0001	1.05 (1.00–1.10)	0.077
Gender of the adolescents												
Male (reference)												
Female	1.12 (1.09–1.15)	<0.0001	1.43 (1.41–1.45)	<0.0001	1.15 (1.10–1.20)	<0.0001	1.43 (1.31–1.57)	<0.0001	1.33 (1.30–1.37)	<0.0001	1.21 (1.18–1.23)	<0.0001
Food insecurity												
None (reference)												
Sometimes or rarely	1.23 (1.20–1.26)	<0.0001	1.18 (1.17–1.20)	<0.0001	1.18 (1.13–1.23)	<0.0001	1.20 (1.07–1.35)	0.002	1.13 (1.10–1.17)	<0.0001	1.17 (1.14–1.20)	<0.0001
Most of time/always	1.32 (1.27–1.37)	<0.0001	1.18 (1.15–1.22)	<0.0001	1.23 (1.14–1.34)	<0.0001	0.99 (0.66–1.51)	0.974	1.19 (1.14–1.25)	<0.0001	1.25 (1.20–1.30)	<0.0001
Bullied												
No (reference)												
Yes	1.24 (1.21–1.27)	<0.0001	1.33 (1.31–1.35)	<0.0001	1.26 (1.20–1.33)	<0.0001	1.30 (1.14–1.48)	<0.0001	1.10 (1.07–1.13)	<0.0001	1.29 (1.26–1.32)	<0.0001
Physically attacked												
No (reference)												
Yes	1.08 (1.05–1.11)	<0.0001	1.11 (1.09–1.13)	<0.0001	1.00 (0.95–1.05)	0.977	1.14 (1.01–1.28)	0.032	1.07 (1.04–1.10)	<0.0001	1.06 (1.04–1.09)	<0.0001
Physical altercations												
No (reference)												
Yes	1.10 (1.07–1.13)	<0.0001	1.05 (1.03–1.06)	<0.0001	1.12 (1.06–1.18)	<0.0001	1.07 (0.97–1.18)	0.201	1.11 (1.08–1.14)	<0.0001	1.13 (1.10–1.16)	<0.0001
Seriously injured												
No (reference)												
Yes	1.20 (1.17–1.23)	<0.0001	1.18 (1.16–1.20)	<0.0001	1.16 (1.11–1.21)	<0.0001	1.14 (1.03–1.25)	0.009	1.27 (1.24–1.31)	<0.0001	1.23 (1.20–1.25)	<0.0001
Peer support												
Sometimes or rarely (reference)												
Never	0.93 (0.91–0.96)	<0.0001	0.96 (0.95–0.98)	<0.0001	1.00 (0.96–1.05)	0.964	0.93 (0.84–1.02)	0.103	0.88 (0.85–0.90)	<0.0001	0.94 (0.92–0.96)	<0.0001
Most of time/always	0.91 (0.88–0.94)	<0.0001	0.95 (0.93–0.97)	<0.0001	0.91 (0.85–0.98)	0.014	0.86 (0.75–0.99)	0.038	0.98 (0.95–1.02)	0.379	1.05 (1.02–1.09)	0.002
Parents check homework												
Never	1.08 (1.04–1.11)	<0.0001	1.06 (1.04–1.08)	<0.0001	1.09 (1.03–1.16)	0.003	1.11 (0.99–1.24)	0.071	1.04 (1.01–1.08)	0.011	1.05 (1.01–1.08)	0.004
Sometimes or rarely	1.04 (1.01–1.07)	0.019	1.03 (1.02–1.05)	<0.0001	0.99 (0.94–1.04)	0.636	1.05 (0.95–1.17)	0.319	1.02 (0.99–1.05)	0.261	1.02 (1.00–1.05)	0.057
Most of time/always (reference)												
Parents understand their child's problems												
Never	1.05 (1.01–1.08)	0.013	1.13 (1.11–1.15)	<0.0001	1.16 (1.09–1.24)	<0.0001	1.14 (0.97–1.35)	0.108	1.15 (1.11–1.19)	<0.0001	1.09 (1.06–1.13)	<0.0001
Sometimes or rarely	1.05 (1.02–1.08)	0.001	1.12 (1.11–1.14)	<0.0001	1.20 (1.14–1.26)	<0.0001	1.17 (1.06–1.29)	0.002	1.08 (1.05–1.12)	<0.0001	1.10 (1.07–1.13)	<0.0001
Most of time/always (reference)												
Parent monitoring												
Never	0.99 (0.96–1.03)	0.617	1.12 (1.10–1.14)	<0.0001	1.08 (1.01–1.16)	0.024	1.07 (0.89–1.28)	0.462	1.05 (1.02–1.09)	0.004	1.06 (1.02–1.09)	0.001
Sometimes or rarely	1.04 (1.01–1.07)	0.005	1.11 (1.09–1.13)	<0.0001	1.13 (1.08–1.19)	<0.0001	1.03 (0.93–1.14)	0.578	1.06 (1.02–1.09)	0.001	1.05 (1.02–1.07)	<0.0001
Most of time/always (reference)												
Miss school without permission												
No (reference)												
Yes	1.10 (1.06–1.15)	<0.0001	1.05 (1.03–1.07)	<0.0001	1.13 (1.05–1.23)	0.002	1.04 (0.88–1.21)	0.661	1.04 (1.00–1.08)	0.031	1.14 (1.11–1.18)	<0.0001
Sedentary activities hours per day												
<1 h (reference)												
1–2 h	1.01 (0.98–1.04)	0.356	1.07 (1.05–1.09)	<0.0001	1.18 (1.12–1.25)	<0.0001	0.99 (0.86–1.14)	0.870	1.00 (0.97–1.04)	0.951	1.04 (1.02–1.07)	0.001
3–4 h	1.07 (1.03–1.11)	<0.0001	1.11 (1.09–1.13)	<0.0001	1.23 (1.15–1.31)	<0.0001	1.06 (0.92–1.22)	0.408	1.09 (1.05–1.13)	<0.0001	1.12 (1.09–1.15)	<0.0001
>4 h	1.09 (1.05–1.13)	<0.0001	1.16 (1.13–1.18)	<0.0001	1.31 (1.23–1.40)	<0.0001	1.13 (0.98–1.30)	0.087	1.15 (1.11–1.19)	<0.0001	1.19 (1.16–1.23)	<0.0001
Adolescent BMI												
Normal weight (reference)												
Overweight	0.99 (0.95–1.04)	0.777	0.99 (0.97–1.01)	0.249	0.94 (0.87–1.03)	0.195	0.90 (0.77–1.06)	0.222	0.99 (0.96–1.03)	0.744	1.04 (1.00–1.07)	0.026
Obesity	0.99 (0.94–1.06)	0.836	0.96 (0.93–0.99)	0.006	0.91 (0.79–1.04)	0.161	0.85 (0.53–1.35)	0.485	1.02 (0.97–1.07)	0.407	1.00 (0.95–1.04)	0.874

aIRR, adjusted incidence rates ratio; BMI, body mass index.

## Discussion

Psychological health problems are among the primary causes of non-fatal illness burdens around the world, yet systematic understandings of their prevalence, disease burden and risk factors at national, regional and global levels is lacking. In this study, we describe national, regional and global prevalence and disease burden of adolescent psychosocial health problems. Globally, our findings suggest that the likelihood of an adolescent experiencing psychosocial health problems increases with age, food insecurity and female gender, as well as among adolescents who experience bullying, physical attacks, serious injury, engagement in physical altercations, sedentary lifestyle, missing school without permission and lack of parental monitoring. The likelihood of experiencing psychosocial health problems was significantly higher among adolescents whose parents did not understand their problems. Surprisingly, psychosocial health problem rates were significantly lower among obese adolescents.

The findings of increased risk of psychosocial health problems among older and female adolescents align with reports from previous studies.^14,21^ According to Lipari and Hedden,^22^ major transitions from childhood to adulthood occur during late adolescence, leaving youth overwhelmed with decisions about their future and vulnerable to psychosocial health problems. Although the reason for the high risk of psychosocial health problems among female adolescents is unclear, biological and social theories could help to explain this. Fluctuations in ovarian hormones, and specifically decreases in oestrogen, have been identified as a potential cause of psychosocial health problems.^23^ The observed association between gender and psychosocial health problems could also be attributable to the high incidence of sexual and gender-based violence worldwide, as well as antenatal and postnatal stress from teenage pregnancies.^24^ Our finding that adolescents who experience food insecurity have higher rates of experiencing psychosocial health problems is consistent with a related study from Canada.^25^ Adolescents who experience food insecurity grapple with feelings of deprivation, which often translate into anxiety and depression. Consistent with previous evidence,^14,26^ adolescents who experience violence or physical injuries (i.e. bullying, physical attacks, serious injury and physical altercations) have a significantly higher risk of experiencing psychosocial health problems. Such traumatic experiences can affect self-esteem and lead to feelings of loneliness, depression and anxiety. Sometimes, these adolescents perceive suicide as the only way to escape further victimisation.^27^

The present study suggests that parental involvement is critical for addressing psychosocial health problems among adolescents, which matches similar findings reported in a related study.^28^ We posit that parental monitoring serves are a powerful tool that allows parents to track changes in their adolescent child's mood, identify distress and assess their mental health, facilitating preventive mechanisms like early remedial interventions. Although the rates of experiencing psychosocial health problems were high among adolescents with a sedentary lifestyle, they were lower among obese adolescents compared with those with a normal BMI. However, this finding is in agreement with a study by Arbour-Nicitopoulos et al,^29^ which showed that adolescent sedentary behaviour significantly increased psychosocial health problems, but found no association between psychosocial health problems and weight status. Female gender, experiencing food insecurity, bullying, physical attacks and serious injury were significantly associated with experiencing psychosocial health problems across all six regions. There was also heterogeneity between regions. Physical altercations were significantly associated with psychosocial health problems in all regions except Europe. Substantial policies and interventions implemented across Europe to reduce and penalise physical altercations could explain this outlier. Notably, the INSPIRE technical package implemented by the European Union could be a contributing factor for the observed associations in this study. Through the INSPIRE technical package, experiences of physical altercations are reduced in childhood even before individuals get to adolescence.^30^

We also identified that participants who had peer support most of the time or always were less likely to experience psychosocial health problems in all regions except the Eastern Mediterranean region. The Eastern Mediterranean region is characterised by high levels of societal insecurity, which commonly affects adolescent mental health.^31,32^ Here, peer support does not offer any significant leverage, as all or most adolescents are struggling. Sedentary behaviour and missing school without permission increased psychosocial health problems across all regions except Europe. Although adolescents without parental monitoring had increased rates of psychosocial health problems in the Americas, South-East Asia, Eastern Mediterranean and Western-Pacific regions, it emerged as insignificant for Africa and Europe. It is worth noting that, although social and family structures in Africa are rapidly evolving, the region continues to rely on extended family and society in the affairs of adolescents. As such, the absence of parental monitoring may not necessarily affect adolescents, as other bodies, such as family, school, religious institutions and general society, step in to detect and resolve issues that could lead to psychosocial health problems.

Given that food insecurity was significantly associated with psychosocial health problems across all six regions, it is imperative for countries to strengthen and expand existing food initiatives (e.g. Ghana's school feeding programme) that aim to provide adequate, safe and nutritious food to in-school adolescents. Our study further highlights the need to create safe spaces in schools to reduce violence or physical injuries, a high-risk factor for adolescent psychosocial health problems. The findings from this study emphasise that establishing school-based programmes and activities that promote peer or parent–adolescent connectedness could be vital to reducing psychosocial health problems among adolescents worldwide. Parents would have to prioritise building healthy relationships with adolescents through consistent interactions and attention to the adolescent's needs. Adolescents would also have to make conscious efforts to maintain their relationship with parents. Our finding that female adolescents are more likely to experience psychological problems underscores the need for governments and states, as well as schools, to pay more attention to the psychological needs of female adolescents.

### Strengths and limitations

The strengths of this study include a large number of adolescents from 68 LMICs and HICs across the six WHO regions, most of which included nationally representative samples. The GSHS implemented standardised methods across surveys, such as sample selection (e.g. school-based), data collection procedures and similarly worded questionnaires, which facilitated comparable assessments of cross-national or regional differences^33^ in psychosocial health phenomenon. The results presented here are obtained using weighted analyses, where the GSHS weighting accounted for the distribution of the population by gender and age. The weighting was used to ensure that the results could be generalised to the entire target population, and not just those who participated in the survey. Therefore, any skewness in the observed data by gender (or age) is unlikely to influence the weighted analysis results.

Findings should also take into account the following limitations. The GSHS measures have limited evidence of reliability and validity across different cultural settings. The use of a self-reported questionnaire is susceptible to social desirability and recall bias. Some adolescents may have had problems in understanding the questionnaire (e.g. poor reading skills), and similarly, parental consent for offspring to participate may have depended on having sufficient literacy skills to read and agree to the consent forms. Because of the sensitive nature of questions on psychosocial health problems, willingness to respond to certain items may have varied depending on sociocultural backgrounds, which could affect the results.^33^ Countries were allowed to use translated versions of the GSHS; therefore, translation into local languages may also have affected the findings,^33^ particularly in cases where local languages may not have words analogous to the original terms used to describe questions related to psychosocial health problems. The study includes data collected over a 13-year period (2003–2015). The period effect, therefore, may have biased the results. Another limitation of our study is that there are likely other residual confounders, such as cultural norms and expectations,^34^ that the study cannot adjust for because of reliance on a secondary data-set. We know that multilevel analysis is centrally concerned with modelling population heterogeneity, both at the population level and geographical level. Because of the unavailability of cluster-level data from several countries, we could not perform multilevel modelling in this context, keeping cluster as a two-level factor. Thus, IRR estimates could be overestimated. Thus, applying multilevel modelling to adjust for cluster effects on outcome variables would be the best suited for data where cluster sampling is adopted and cluster-level information is available. Despite these limitations, this study's findings gathered from such a large, globally representative sample provide insights that are generally inaccessible by smaller data-sets, and adds valuable nuance to the mental health literature.

In conclusion, adolescents’ psychosocial health problems are influenced by several factors, including age, food insecurity, being female, experiencing bullying, physical attacks, serious injuries, physical altercations, sedentary lifestyle, missing school without permission, lacking parental monitoring, having guardians who do not understand their problems and weight status. We further conclude that there are regional variations in the risk of adolescent psychosocial health problems. Policies and interventions to reduce adolescents’ psychosocial health problems should increase focus on parents and female adolescents. Greater attention should be given to creating more safe spaces for adolescents at school, as well as enforcing strict policies on bullying and other forms of violence that serve as risk factors to experiencing psychosocial health problems.

## Supporting information

## Data Availability

The data that support the findings of this study are openly available (https://extranet.who.int/ncdsmicrodata/index.php/catalog/gshs/?page=1&ps=15&repo=GSHS). The authors do not hold the rights to share this data-set.
